# Report of the Diseases of Edinburgh for February, 1810

**Published:** 1810-04

**Authors:** John Roberton


### *Report of the Diseases of Edinburgh for February*, 1810. By JOHN ROBERTON, M. D.

The mildness of the weather, at the commencement of this month, has, I believe, scarcely ever been equalled in our northern latitude at the same period. In consequence of this, vegetation made considerable progress, and insects of various kinds, whose existence can only be expected during the summer months, appeared in various places. In a week, however, we were again plunged in the very depth of winter. Frost and snow prevailed, and the first of these continued gradually to increase in severity. Additional and frequent, though not heavy falls of snow, occasionally accompanied it; and the weather became excessively cold. Before the termination of the month, we had also frequent falls of snow, which, however, were neither very heavy, nor did they continue long on the ground. The weather toward the end of the month was alternately soft and frosty, with frequent most tremendous hurricanes.

The barometer, till near the end of the month, was, in general, rather high, but then it fell considerably.

The thermometer, during the first third of it, stood, during the day, from 45 to 50, and some days it was even above 50. During the night, however, it sunk to near the freezing point. From this, till within a few days of the termination of the month, it was generally below the freezing point, and for several days together was seldom less than 10 degrees below it. During the soft days, near the end of the month, it of course rose considerably.

The diseases which have prevailed, have been numerous, but in general not very destructive. To those of last month, most of which still prevail, there have been added chin-cough, measles, and bowel complaints; and the cases of inflammation of the lungs, &c. and those of catarrh and cynanche tonsillaris, have become both more general and more severe. This increase of severity, in some of these these complaints, may easily be accounted for. It is very common, in this part of the world, very early in the year, for the weather for a short period to become mild and agreeable. This induces a great proportion of the thoughtless part of the community to alter their dress, by too early taking off a part of their winter clothing. This annual mistake, subjects numbers to complaints, which, were they to recollect for a moment, the very unfavourable sort of climate in which they exist, would probably never affect them. Thus some very severe cases of catarrh are frequently to be met with during the spring months, and many of a consumptive habit, from this circumstance alone, have their lungs affected, which, with greater care and attention, might have been prevented for a long period, if not for the remaining years of their lives.

I may mention, also, that the cases of cynanche tonsillaris are, in general, considerably aggravated since last month. There are also various cases, independently of the throat being affected, in which considerable feverishness exists; the mouth becomes inflamed, and unless speedily checked by confinement to the house, by repeated doses of physic, gargles, &c. it proceeds to ulceration, and proves exceedingly troublesome to the patient, and in many, especially those advanced in life, some of their teeth have fallen out. In such cases, however, even when permitted to proceed to the greatest pitch of severity, I remove them in a short period by frequently washing the mouth with port wine and water, and by administering repeated smart doses of physic.

The cases of chin-cough do not seem to be numerous, nor do the measles prevail very extensively. Both of these diseases, however, have, in various instances under my observation, affected three or four of the children, (for among them they are chiefly to be met with) of one family. I have found the most successful practice in the chin-cough, to be that of instantly, on the appearance of the first symptoms, putting the child to bed, and, during the first few days, preserving the body in a very equal and rather elevated temperature. This has, in general, seemed greatly to moderate the symptoms.

In such cases of the measles as have come under my observation, I have found the greatest benefit arise from the body being preserved in a temperature of about 60°. That, with the application of two or three leeches to the temples, on the first appearance of the symptoms, has been conspicuously beneficial.

Inflammation

Inflammation of the lungs, in particular, has appeared more frequently than at any period during the winter, and in greater severity. In several cases, I have found it necessary to order venæsection once every day for eight or ten days, even till the patient fainted at each time. In several cases, where the utmost activity of practice was persevered in, the patients have died. Some of their bodies were opened after death, and the adhesions between the lungs and pleura in some, were inconceivably strong, and existed at every point where the lungs could be brought in contact with the pleura. In others, there was an immense quantity of a sort of purulent matter secreted in the thorax.

The bowel complaints progressively became worse from the commencement till the termination of the month. This has, in almost every instance, been attended with feverishness; and the acute pains in the bowels which many suffered, were indescribably severe. In general, however, two or three doses of physic entirely removed this complaint; but in others, I have found it necessary, daily to employ emetics, and physic alternately, for more than a week.

*Princes Street.*

